# Co-existence of multiple distinct lineages in *Vibrio parahaemolyticus* serotype O4:K12

**DOI:** 10.1099/mgen.0.000287

**Published:** 2020-10-04

**Authors:** Lin Zhao, Hongyou Chen, Xavier Didelot, Zhenpeng Li, Yinghui Li, Meiling Chen, Yu Du, Hongqun Zhao, Jie Li, Qinghua Hu, Biao Kan, Min Chen, Bo Pang

**Affiliations:** ^1^​ State Key Laboratory of Infectious Disease Prevention and Control, National Institute for Communicable Disease Control and Prevention, China CDC 155, Changbai Road, Changping, Beijing, PR China; ^2^​ Division of Pathogen Detection, Shanghai Municipal Center for Disease Control and Prevention 1380, Zhongshan West Road, Changning District, Shanghai, PR China; ^3^​ School of Life Sciences and Department of Statistics, University of Warwick, Coventry, UK; ^4^​ Collaborative Innovation Center for Diagnosis and Treatment of Infectious Diseases, Hangzhou, PR China; ^5^​ Microbiology Laboratory, Shenzhen Center for Disease Control and Prevention, Shenzhen, PR China; ^6^​ Chunshu Community Healthcare Center, Xicheng District, Beijing, PR China; ^‡^​ Present address: MGI, BGI-Shenzhen, BeiShan Industrial Zone, Yantian District, Shenzhen, Guangdong, PR China

**Keywords:** *V. parahaemolyticus*, population structure, recombination, pathogenic island, MLST, whole genome sequencing

## Abstract

*
Vibrio parahaemolyticus* is an important cause of foodborne gastroenteritis globally. Thermostable direct haemolysin (TDH) and the TDH-related haemolysin are the two key virulence factors in *V. parahaemolyticus. Vibrio* pathogenicity islands harbour the genes encoding these two haemolysins. The serotyping of *V. parahaemolyticus* is based on the combination of O and K antigens. Frequent recombination has been observed in *V. parahaemolyticus *, including in the genomic regions encoding the O and K antigens. *V. parahaemolyticus* serotype O4:K12 has caused gastroenteritis outbreaks in the USA and Spain. Recently, outbreaks caused by this serotype of *V. parahaemolyticus* have been reported in China. However, the relationships among this serotype of *V. parahaemolyticus* strains isolated in different regions have not been addressed. Here, we investigated the genome variation of the *V. parahaemolyticus* serotype O4:K12 using the whole-genome sequences of 29 isolates. We determined five distinct lineages in this strain collection. We observed frequent recombination among different lineages. In contrast, little recombination was observed within each individual lineage. We showed that the lineage of this serotype of *V. parahaemolyticus* isolated in America was different from those isolated in Asia and identified genes that exclusively existed in the strains isolated in America. Pan-genome analysis showed that strain-specific and cluster-specific genes were mostly located in the genomic islands. Pan-genome analysis also showed that the vast majority of the accessory genes in the O4:K12 serotype of *V. parahaemolyticus* were acquired from within the genus *Vibrio *. Hence, we have shown that multiple distinct lineages exist in *V. parahaemolyticus* serotype O4:K12 and have provided more evidence about the gene segregation found in *V. parahaemolyticus* isolated in different continents.

## Data Summary

All the sequencing data have been deposited in GenBank under BioProject ID no. PRJNA515151 (www.ncbi.nlm.nih.gov/bioproject/PRJNA515151). All the supporting data have been provided through supplementary data files. Scripts were submitted to GitHub (https://github.com/duominuolin/seq_aln_map).

**Impact Statement**

*
Vibrio parahaemolyticus* is an important cause of foodborne gastroenteritis globally. *V. parahaemolyticus* serotype O4:K12 has caused gastroenteritis outbreaks in the USA and Spain. Recently, outbreaks caused by this serotype of *V. parahaemolyticus* have been reported in China. However, variation among this serotype of *V. parahaemolyticus* isolated in China and the relationship between the *V. parahaemolyticus* serotype O4:K12 isolated in different continents remained to be illuminated. In this study, we investigated the genomic variation of the *V. parahaemolyticus* serotype O4:K12 by integrating newly sequenced genomes and those publicly available. We observed that strains isolated in different continents formed separate lineages. We showed different levels of recombination between and within different lineages. We also showed that the vast majority of the accessory genes in the O4:K12 serotype of *V. parahaemolyticus* were acquired from within the genus *Vibrio* by pan-genome analysis. We showed that serotyping is not capable of reflecting the variation in the O4:K12 serotype of *V. parahaemolyticus *. Our results provide complementary information to previous studies of the genomic variation characteristics of the *V. parahaemolyticus* serotype O4:K12.

## Introduction

*Vibrio parahaemolyticus *, a Gram-negative halophilic bacterium, is recognized as an important cause of foodborne gastroenteritis globally. The thermostable direct haemolysin (TDH) and the TDH-related haemolysin (TRH) are the two key virulence factors in *V. parahaemolyticus* [1–3]. Pathogenic *V. parahaemolyticus* typically produce at least one of these two haemolysins. The *tdh* and *trh* genes, which encode TDH and TRH, respectively, are harboured on *Vibrio* pathogenicity islands (VPaIs). Besides these two VPaIs, several other VPaIs have been determined and some of these VPaIs have been considered to give advantages to the bacterium that carries them [4]. *V. parahaemolyticus* also carries type three secretion systems (TTSSs) that contribute to the interaction between the bacterium and host [5]. Two kinds of TTSS, TTSS1 and TTSS2, have been identified so far in *V. parahaemolyticus* [5]. TTSS2 can be further classified into TTSS2α and TTSS2β [4, 6–8]. In pathogenic *V. parahaemolyticus *, TTSS2α and TTSS2β are genetically linked to *tdh1/tdh2* and *trh*, respectively [4, 7, 9]. The VPaI-7 that harbour TTSS2 variants and their corresponding haemolysin genes are named VPaIα, VPaIβ and VPaIγ systematically [8].

The serotyping scheme of *V. parahaemolyticus* is based on the combination of O and K antigens [10]. *V. parahaemolyticus* is a bacterium that experiences frequent recombination [11, 12], and the recombination in the region that surrounds the O- and K-antigen-encoding gene cluster contributes to the serotype conversion of this bacterium [9]. *V. parahaemolyticus* also showed segregation of variation between different oceans and this is consistent with conventional population genetic models of limited dispersal between gene pools [12]. The *V. parahaemolyticus* serotype O4:K12 has caused gastroenteritis outbreaks in the Pacific Northwest region in the USA in around 1997 [13], and on the Atlantic coasts of the USA and Spain in 2012 [14]. These strains were positive for both TDH and TRH, and were found to belong to multilocus sequence type 36 (ST36) [14]. This serotype of *V. parahaemolyticus* was also isolated in Vietnam [15] and Chile [16]. In China, the *V. parahaemolyticus* serotype O4:K12 caused gastroenteritis outbreaks in 2006, 2010, 2011 and 2014 in Shanghai. This serotype of *V. parahaemolyticus* was also isolated in Shenzhen (China) in 2012 [17]. However, variation among this serotype of *V. parahaemolyticus* isolated in China and the relationship among the *V. parahaemolyticus* serotype O4:K12 isolated in different regions has not been investigated on a broader scale.

In the present study, we addressed the questions above by whole-genome sequencing. We showed that multiple distinct lineages exist within this O4:K12 serotype of *V. parahaemolyticus *. The serotype O4:K12 *V. parahaemolyticus* isolated in Asia are phylogenetically divergent from those isolated in America. The pan-genome analysis showed that the genomic differences between Asian and American strains clustered mainly in genome islands.

## Methods

### Strain collection

A total of 25 *V *. *parahaemolyticus* serotype O4:K12 strains isolated from patients between 2006 and 2014 during routine surveillance in China were selected for analysis. The serotype was confirmed by agglutination with a specific antiserum kit (Tianjin Biochip).

### Strain culture and DNA extraction

All the 25 strains sequenced in this study were recovered on Luria–Bertani (LB) agar from storage (in a −80 °C freezer). A single colony was transferred to LB broth with 3 % NaCl and was incubated at 37 °C with shaking at 200 r.p.m. Genomic DNA was extracted from overnight cultures with a Wizard genomic DNA purification kit (Promega) according to the manufacturer's instructions.

### Whole-genome sequencing

Whole-genome sequencing of 25 genomes was performed on an Illumina HiSeq 2000 instrument with a 500 bp insertion fragment library (SinoGenoMax). All the data have been submitted to GenBank under BioProject ID no. PRJNA515151.

### Genome assembly and identification of single nucleotide variants (SNVs)

Short reads were assembled *de novo* into contigs and scaffolds using SPAdes (V3.7) [18]. MuMMER (V3.0) was used to compile a whole-genome alignment for all of the genomes used in this study [19]. The whole-genome sequence of RIMD2210633 [7] was used as the reference to call SNVs in MuMMER. Any SNV with a quality score lower than 30 was excluded.

### Phylogenetic and comparative genomics analysis

In addition to the 25 genomes sequenced in this study, as described above, 112 representative genomes from a previous study [12] were also included in the phylogenetic analysis. The neighbour-joining tree was constructed using NJtree (http://treesoft.sourceforge.net/njtree.shtml) (now called TreeBeST – http://treesoft.sourceforge.net/treebest.shtml). We used ClonalFrameML v1.11 [20] to analyse the clonal relationships and the effect of recombination in *V. parahaemolyticus* serotype O4:K12. Prophages, insertion sequence elements and repetitive sequences were identified in the reference genome RIMD2210633. These regions were marked in the alignment and deleted in the phylogenetic analyses of the 29 *V *. *parahaemolyticus* serotype O4:K12 and 22 ST813 genomes. The maximum-likelihood (ML) trees of the 29 *V *. *parahaemolyticus* serotype O4:K12 and the 22 ST813 *V. parahaemolyticus* were built using raxmlHPC with 100 bootstraps [21].

### Principal component analysis (PCA)

A matrix (M×N) was constructed based on the presence or absence of accessory genes (N) of each strain (M). If an accessory gene existed in a strain, the corresponding position of matrix was recorded as 1, otherwise it was recorded as 0. PCA was carried out based on the presence of common (5–95 % prevalence) accessory genes. The prcomp function in R language was used to do PCA computing.

### Distribution of known VPaIs in the O4:K12 *V. parahaemolyticus*

To describe the detailed variations of genome islands in the tested *V. parahaemolyticus* serotype O4:K12, we compared these genomes with known genome islands found in *V. parahaemolyticus* (Table S1, available with the online version of this article) using BLASTn with an identity >80 % and an *e* value of <1×10^−5^. The visualization of the distribution of these VPaIs was implemented with iTOL [22].

### Pan-genome analysis of the O4:K12 *V. parahaemolyticus*

The assemblies were annotated using Prokka (1.12) [23]. The pan-genome matrix was calculated using Roary with an identity set as 95 % [24]. The heat map of the presence and absence of accessory genes was constructed with the gplots 2.11.0 (https://cran.r-project.org/web/packages/gplots/index.html) package of R.

### Antimicrobial resistance gene detection

ResFinder (https://cge.cbs.dtu.dk/services/ResFinder/, accessed 01/04/2019) was used to identify acquired antimicrobial-resistance genes.

## Results

### ST analysis of the 29 genomes of *V. parahaemolyticus* O4:K12

In order to explore the population diversity of *V. parahaemolyticus* serotype O4:K12, we performed multilocus sequence typing (MLST) analysis of the 25 *V *. *parahaemolyticus* serotype O4:K12 isolated in routine surveillance in China between 2006 and 2014 (Table S2). We also obtained the draft genome sequences of another four *V *. *parahaemolyticus* serotype O4:K12 strains from a previous study via GenBank. The sequences of the seven housekeeping genes in the 29 *V *. *parahaemolyticus* were extracted from the assemblies. Query of allelic profiles against the PubMLST database (http://pubmlst.org/vparahaemolyticus/) revealed six STs in these 29 strains, including two new STs. These six STs were: ST36 (*n*=1, clonal complex 36), ST59 (*n*=1, clonal complex 36), ST933 (*n*=1, singleton), ST2009 (*n*=1), ST2010 (*n*=1) and ST813 (*n*=22, singleton). The new STs (ST2009 and ST2010) were based on the identification of two new alleles (*recA* allele 350 and *pntA* allele 241) in strains vp100057 (ST2009) and vp110079 (ST2010), respectively (Table S3). These STs were scattered in the tree built by GrapeTree (Fig. S1) [25]. We could not determine the ST of one strain isolated in Japan (S058, accession no. AWLF00000000) and one strain isolated in the USA (S037, accession no. AWLY00000000) because one of the seven housekeeping genes was incomplete in the assemblies.

### Phylogenetic analysis of *V. parahaemolyticus* O4:K12 with whole-genome sequencing

**Fig. 1.**
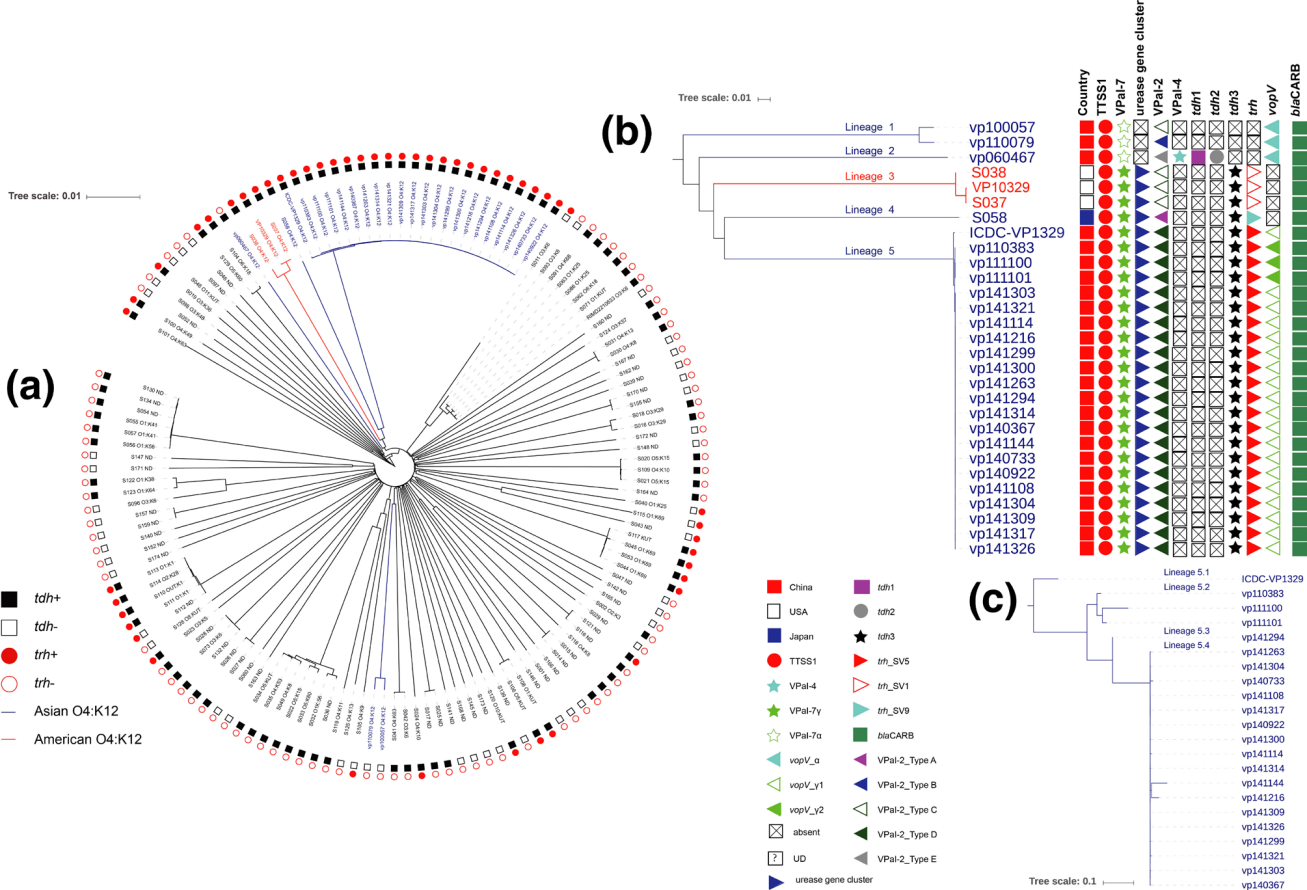
(a) Neighbour-joining tree of 137 *V *. *parahaemolyticus* genomes. The O4:K12 *V. parahaemolyticus* isolated in Asia are shown in dark blue and those isolated in America are in red. The serotypes of the strains, and the presence of *tdh* and *trh* are also labelled. (b, c) ML tree constructed on the SNVs identified in the non-repetitive, non-recombinant core-genome of the 29 O4:K12 (Fig. 1b) and 22 ST813 (Fig. 1c) *V. parahaemolyticus *. The strain information and the distribution of TTSS1, VPaIs, the *ure* gene cluster, *tdh*, *trh*, *vopV* and antibiotic-resistance genes have been annotated on the right of the ML tree using iTOL [22].

**Fig. 2.**
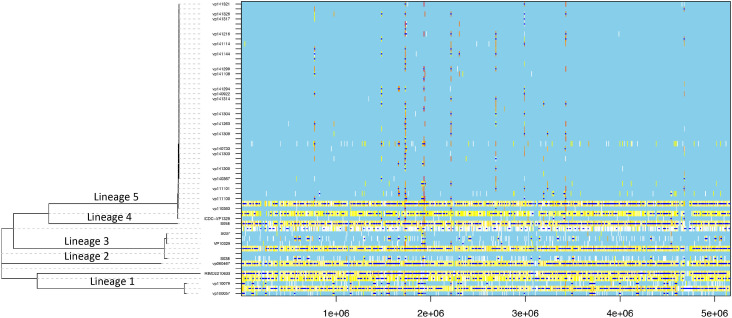
Results of the ClonalFrameML analysis of the O3:K6 RIMD2210633 and the 29 O4:K12 *V. parahaemolyticus* genomes. The tree on the left is the clonal genealogy inferred by ClonalFrameML after accounting for the effect of recombination. For each branch of this tree there is a row in the heatmap on the right, which shows where polymorphisms have been found (coloured white if compatible with the tree, otherwise coloured yellow to red according to the increasing level of incompatibility) and where recombination events have been inferred (dark blue horizontal bars).

In order to explore the phylogenetic position of *V. parahaemolyticus* serotype O4:K12 in the *V. parahaemolyticus* population, the 25 collected *V *. *parahaemolyticus* serotype O4:K12 strains were subjected to whole-genome sequencing and were integrated with the 112 representative genomes of various serotypes of *V. parahaemolyticus* from a previous study [12]. In total, 29 *V *. *parahaemolyticus* serotype O4:K12 were included. We constructed a neighbour-joining phylogeny (Fig. 1a) of 137 *V *. *parahaemolyticus* genomes (Table S2) by using 232 776 SNVs, with reference to the complete genome sequence of strain RIMD2210633 [7], excluding SNVs in prophage, insertion sequence elements, repetitive sequence and recombination regions. The 29 *V *. *parahaemolyticus* serotype O4:K12 formed two distant clusters in the neighbour-joining tree (Fig. 1a). In order to further investigate the relationship among these 29 *V *. *parahaemolyticus* serotype O4:K12, we constructed a ML phylogeny of these strains with ClonalFrameML [20] by using 161 159 SNVs in the genomes, with reference to the complete genome sequence of strain RIMD2210633. The ClonalFrameML analysis showed that on the long branches of the phylogeny there was often more than 50 % of recombinant sites, whereas amongst the cluster of 22 closely related genomes, there was less than 5 % of recombinant sites (Fig. 2). This pattern is consistent with the clonal relationship between the 22 genomes. ClonalFrameML estimated that overall recombination is R/theta=0.46 times as frequent as mutation. Recombinant fragments were on average delta=140 bp long, and carried polymorphism on a proportion nu=0.027 of sites. Therefore, the relative effect of recombination versus mutation is r/m=(R/theta)×delta×nu=1.74. We arbitrarily determined five lineages in these 29 genomes by eye (Fig. 1b). Lineage 1 consisted of two strains isolated in 2010 and 2011 in China (*tdh* ^-^ *trh* ^-^); lineage 2 only consisted of one strain isolated in 2006 in China (*tdh* ^+^ *trh* ^-^); lineage 3 consisted of three strains isolated in the USA (*tdh* ^+^ *trh* ^+^); lineage 4 only consisted of one strain isolated in Japan in 1970 (*tdh* ^+^ *trh* ^+^) and lineage 5 consisted of 22 strains isolated between 2011 and 2014 in China (*tdh* ^+^ *trh* ^+^) (Fig. 1b). All the 22 strains in lineage 5 were ST813. In order to investigate the diversity of the ST813 *V. parahaemolyticus* serotype O4:K12 strains, we constructed a ML phylogeny of these strains by using 552 SNVs with the method mentioned above (Fig. 1c). We arbitrarily divided these 22 ST813 strains into four sub-lineages. Lineage 5.1 only consisted of one strain isolated in 2012, lineage 5.2 consisted of three strains isolated in 2011, lineage 5.3 consisted one strain isolated in 2014 and lineage 5.4 consisted of 18 strains isolated in 2014.

### Distribution of genome islands in *V. parahaemolyticus* serotype O4:K12

We examined the distribution of VPaIs (Table S1), from VPaI-1 to VPaI-7 [4], in the O4:K12 strains. VPaI-1, VPaI-3, VPaI-5 and VPaI-6 were not detected in the O4:K12 strains (Fig. 1b). The VPaI-4 variant, which has coverage of 77 % of the prototype, was only detected in the single strain in lineage 2. There was a segment replacement in this variant compared to VPaI-4 in RIMD2210633 [7] (Fig. S2). Moreover, VPaI-4 was in chromosome 1 in RIMD2210633, while the VPaI-4 variant was in chromosome 2 in this strain. Five alternative types of VPaI-2 were identified in test O4:K12 strains (Table S4). The strain in lineage 4 harboured type-A VPaI-2. All strains in lineage 5 harboured type-E VPaI-2. All the three strains in lineage 3 and one strain in lineage 1, which was isolated in 2010 in China, harboured type-C VPaI-2. One strain in lineage 2 and one strain in lineage 1 harboured type-D and type-B VPaI-2, respectively. VPaI-7 also has been known as VPaI [8]. For systematic reason, we adopted the name VPaI-7 throughout this paper. VPaI-7α was detected in the strains in lineage 1 and 2. VPaI-7γ was detected in the strains in lineage 3, 4 and 5 (Fig. 1).

TTSS1 was detected in all the 29 O4:K12 genomes. TTSS1 in lineage 3 strains showed 90 % coverage of the TTSS1 in RIMD2210633, while TTSS1 in the remaining strains was intact. The urease gene cluster and *trh* were detected in all O4:K12 genomes except for strains in lineage 1 and 2. In total, three types of *trh* gene were detected. SV1, SV9 and SV5 types of *trh* gene were detected in lineage 3, lineage 4 and lineage 5, respectively (Table S4, Fig. 1b) [26]. TTSS2α was detected in strains in lineage 1 and lineage 2. TTSS2β was detected in strains in lineage 3, lineage 4 and lineage 5. The *vopV* gene in three strains in lineage 5 showed almost 100 % similarity to that in MAVP-QPI [8], while the remaining ones showed around 90 % similarity (Fig. S3, Table S4). The *tdh* gene was detected in all O4:K12 strains except for those in lineage 1. The *tdh*1 and *tdh*2 genes were detected in lineage 2, while the *tdh*3 gene was detected in lineage 3, lineage 4 and lineage 5 (Fig. 1b).

### Core-genome and pan-genome analysis

The core-genome and pan-genome of the 29 O4:K12 *V. parahaemolyticus* contained 3866 and 6312 ORFs, respectively. We then divided the 29 O4:K12 *V. parahaemolyticus* into an Asia group and an America group based on the results of a previous study [12]. The core-genome of the Asia group and the America group included 3985 and 4382 ORFs, respectively, while the pan-genome of these two groups contained 5982 and 4722 ORFs, respectively.

**Fig. 3.**
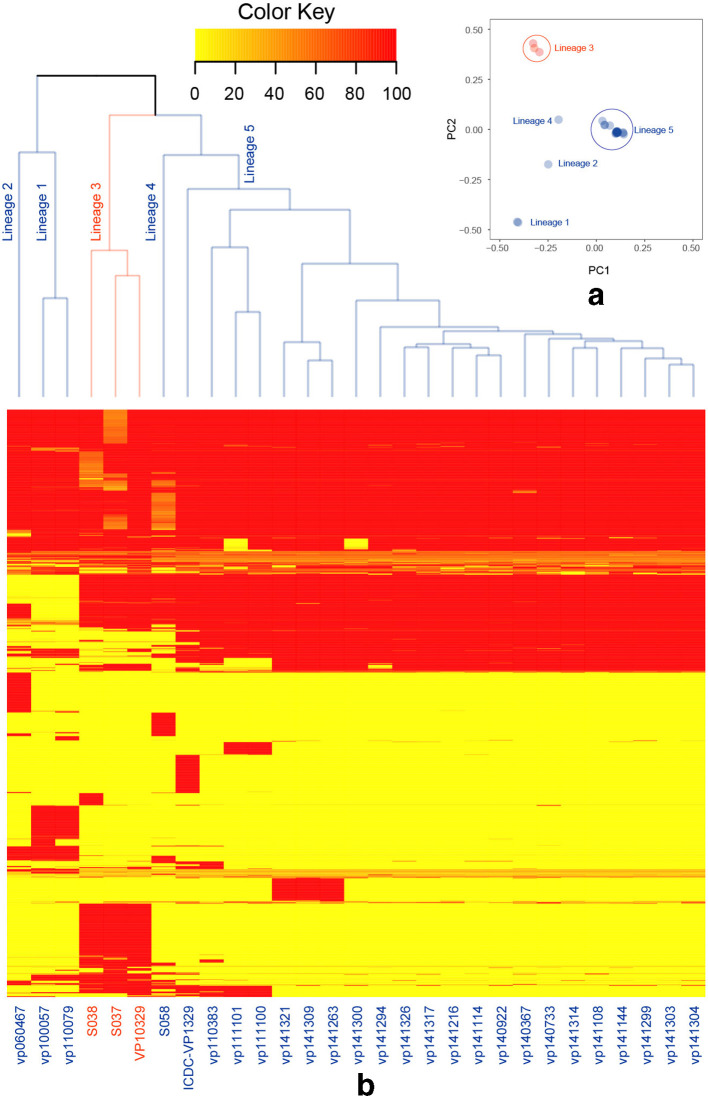
(a) PCA based on the presence of common (5–95 % prevalence) accessory genes in the 29 O4:K12 *V. parahaemolyticus *. The plot displays the relationship between the strains graphically. PCA was carried out by using the strains×genes matrix as input and the plot was displayed by using the scores of PCA results. Strains isolated from North America are displayed as red circles, while strains isolated from Asia are displayed as blue circles. The first two components explained 60.09 % of the variance. The first component explained 38.45 % of the variance, while the second component explained 21.64 % of the variance. (b) Heat map of the presence of accessory genes in the 29 O4:K12 *V. parahaemolyticus *. The dendrogram above the heat map is the clustering of the 29 strains based on the presence of genes.

In order to explore the diversity of O4:K12 *V. parahaemolyticus *, we also investigated the pan-genome of the 29 genomes. We focused on the segments that included at least five continuous ORFs. We divided these segments into strain-specific (SS) (present only in one strain) and cluster-specific (CS) (present in two or more strains) segments. In total, we found 21 SS segments and 25 CS segments (Table S5, Fig. 3). Fourteen of these twenty-one SS segments were similar to the sequences of genomic islands and phages (Table S5). We presumed these segments were lateral gene transfer (LGT) related. Thirteen of these fourteen segments showed best hits to the sequences in *Vibrio *. Among the remaining seven SS segments, two were previously reported, three encoded hypothetical proteins and two were not reported in *V. parahaemolyticus* previously. Similarly, 16 of the 25 CS segments were LGT related and 4 of these 16 segments were similar to the plasmid sequences (Table S5). Fifteen of these sixteen LGT-related segments were similar to the sequences in *Vibrio *. A total of five CS segments (Table S5) were found only in the sequenced *V. parahaemolyticus* O4:K12 isolated in the USA to date and they were related to 12 genome islands in VP10329, which was isolated in the USA [27].

## Discussion

*V. parahaemolyticus* represents a population of high diversity and frequent recombination [12]. The *V. parahaemolyticus* serotype O4:K12 is one of the clonal sub-populations of the diverse *V. parahaemolyticus* population in the Pacific Northwest of the USA [28]. In this study, we showed that five distinct lineages existed in this serotype of *V. parahaemolyticus* and they were dispersed in the phylogenetic tree (Fig. 1a). Moreover, these strains exhibited six distinct and divergent STs. ClonalFrameML identified significant recombination in the 29 O4:K12 *V. parahaemolyticus* genomes, with a relative effect of recombination versus mutation of r/m=1.74. All of these results suggested that the *V. parahaemolyticus* serotype O4:K12 included multiple lineages. The strains isolated in the USA formed a single independent lineage. In the phylogeny, the Asia lineages were remotely related to that lineage which caused outbreaks both in the USA and Spain [13, 14, 29]. Furthermore, we observed CS segments in strains isolated in America. These CS segments were linked to genomic islands in the *V. parahaemolyticus* genomes, which supports the limited dispersal of genes in the gene pools of *V. parahaemolyticus* isolated in America [12] [30]. Among the Asian lineages, the Japanese lineage was far away from any of the three divergent China lineages in the phylogeny. The PCA on accessory gene content clearly distinguished the strains isolated in America and Asia. In the pan-genome analysis, more than half of the SS and CS segments were LGT related. The majority of these LGT-related segments were found in the *Vibrio* sequences deposited in GenBank to date, but some did not align to any sequences in GenBank. This is different from the situation in *Klebsiella pneumoniae *, which can acquire accessory genes from a wide range of bacterial taxa [31]. The SS and CS segment analysis results illustrate the frequent recombination in these strains and their capability of integrating new genes.

Serotyping of *V. parahaemolyticus* is based on the somatic O antigen and the capsular polysaccharide K antigen [10]. The *dgkA* and *gmhD* genes flank the O antigen determinant in *V. parahaemolyticus* [32]. Traditionally, 13 kinds of O antigens and 71 kinds of K antigens have been identified [33]. However, *V. parahaemolyticus* with untypeable and new O or K antigens have been frequently isolated [10, 34, 35]. Therefore, further new O and K antigens could be expected in *V. parahaemolyticus* in the future. Comparison of the genome of O3:K6 and O4:K68 showed 94 % of the SNVs between these two genomes were within the region surrounding the O- and K-antigen-encoding gene cluster, which suggested a possible recombination event [9]. In addition, sequence comparison of determinants of 13 *V *. *parahaemolyticus* O antigens revealed frequent horizontal gene transfer and recombination-mediated gene replacement in this region [36]. Thus, genetically closely related *V. parahaemolyticus* could show divergent serotypes and vice versa. In our study, all the 29 *V *. *parahaemolyticus* strains belong to serotype O4:K12 according to the antiserum agglutination test. Moreover, they showed identical O- and K-antigen-encoding sequences. However, these 29 *V *. *parahaemolyticus* serotype O4:K12 strains formed multiple distinct lineages. This meant that these strains did not have a most-recent common ancestor. The reason that these diverse strains exhibited the same O and K antigen phenotype might be attributed to the acquisition of identical O- and K-antigen-encoding sequences in different events, which is similar to the situation observed in O1 serogroup *Vibrio cholerae* [37].

## Data Bibliography

1. Bo Pang, GenBank, PRJNA515151.

## Supplementary Data

Supplementary material 1

Supplementary material 2
