# Supplementary material for: Co-existence of multiple distinct lineages in Vibrio parahaemolyticus serotype O4:K12
Source: Microb Genom. 2020 Oct 4;6(12):mgen000287. doi: 10.1099/mgen.0.000287 (PMC8116679; doi:10.1099/mgen.0.000287)

Figure S1 GrapeTree based on the MLST result of *V. parahaemolyticus* in the pubMLST database. The O4:K12 *V. parahaemolyticus* were labeled with red circles. The isolation regions and STs of these strains were labeled in different colors respectively.

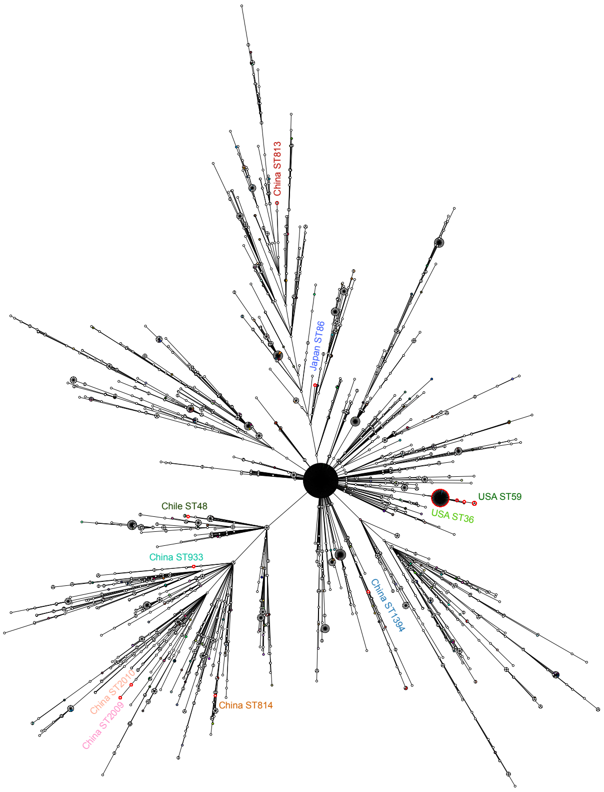

Figure S2 Comparison of VPaI-4 and the VPaI-4 variant. The sequence of VPaI-4 in RIMD2210633 and the VPaI-4 variant in vp060467 were compared and visualized using an in-house Perl script (SVG-2.78) https://github.com/duominuolin/seq_aln_map/tree/master/bin/draw_seq_maps.pl.

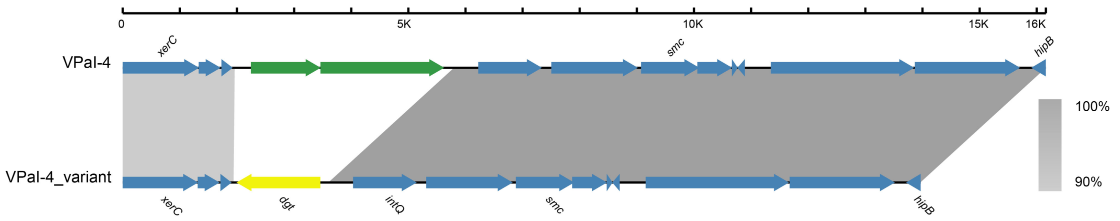

Figure S3 Comparison of the VPaI-7γ_vp141263 (VPaI-7γ in vp141263) and MAVP-QPI (VPaI-7γ in MAVP-Q). (A) Linear comparison of the compositions of MAVP-QPI and VPaI-7γ_vp141263. The *tdh* and *trh* were in red and yellow respectively. (B) Comparison of *vopV*_γ1 (*vopV* gene in MAVP-QPI) and *vopV*_γ2 (*vopV* in vp141263). The repetitive sequences with an identity below 88% were ignored. (C) Comparison of *vopV*_γ1 (*vopV* gene in MAVP-QPI) and *vopV*_γ2 (*vopV* in vp141263). Only the repetitive sequences with the maximum length were kept for illustration of the comparison. An in-house Perl script (SVG-2.78) was used to visualize the VPaI-7 elements in MAVP-Q and vp141263 (Fig.S3A). BLASTn were used to do the alignment (e value > 1e-5, identity > 99%). The Easyfig program (http://mjsull.github.io/Easyfig/files.html, V2.2.3) was used for visualization of the comparison between *vopV* in MAVP-QPI and vp141263 (Figure S3B and Figure S3C).

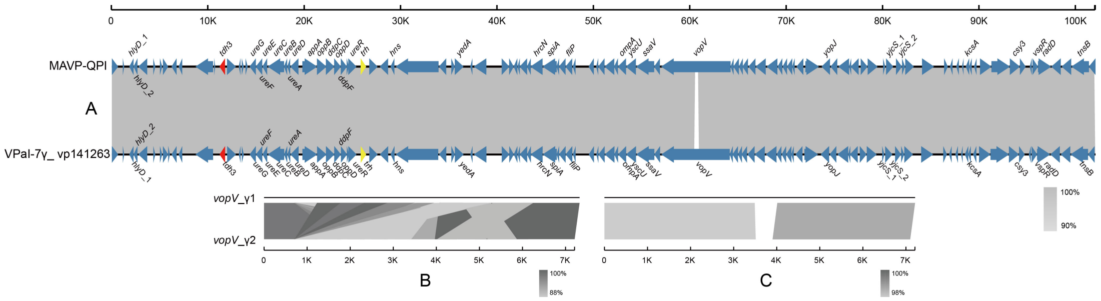

Supplement: Supplementary material 1 [file mgen-6-287-s001.pdf]
